# A polygenic score associated with fracture risk in breast cancer patients treated with aromatase inhibitors

**DOI:** 10.1038/s41523-024-00615-9

**Published:** 2024-01-20

**Authors:** Christine Hook, Udit Chatterjee, Haiyang Sheng, Qianqian Zhu, Timothy Robinson, Janise M. Roh, Cecile A. Laurent, Catherine Lee, Jennifer Delmerico, Joan C. Lo, Christine B. Ambrosone, Lawrence H. Kushi, Marilyn L. Kwan, Song Yao

**Affiliations:** 1 Jacobs School of Medicine and Biomedical Sciences, University at Buffalo, Buffalo, NY USA; 2 Department of Cancer Prevention and Control, Roswell Park Comprehensive Cancer Center, Buffalo, NY USA; 3 Department of Biostatistics, University at Buffalo, Buffalo, NY USA; 4 Department of Biostatistics and Bioinformatics, Roswell Park Comprehensive Cancer Center, Buffalo, NY USA; 5 Population Health Sciences, Bristol Medical School, University of Bristol, Bristol, UK; 6 Division of Research, Kaiser Permanente Northern California, Oakland, CA USA

**Keywords:** Risk factors, Translational research

## Abstract

Identifying women at high risk of osteoporotic fracture from aromatase inhibitor (AI) therapy for breast cancer is largely based on known risk factors for healthy postmenopausal women, which might not accurately reflect the risk in breast cancer patients post-AI therapy. To determine whether a polygenic score associated with fracture in healthy women is also significant in women treated with AIs for breast cancer, we used data from a prospective observational cohort of 2152 women diagnosed with hormonal receptor positive breast cancer treated with AIs as the initial endocrine therapy and examined a polygenic score of heel quantitative ultrasound speed of sound (gSOS) in relation to incident osteoporotic fracture after AI therapy during a median 6.1 years of follow up after AI initiation. In multivariable models, patients with the second and third highest tertiles (T) versus the lowest tertile of gSOS had significantly lower risk of fracture (T2: adjusted HR = 0.61, 95% CI: 0.46-0.80; T3: adjusted HR = 0.53, 95% CI: 0.40-0.70). The lower risk of fracture in patients with the highest tertile of gSOS remained significant after further adjustment for BMD at the hip (T3: adjusted HR = 0.62, 95% CI: 0.42-0.91). In conclusion, our analysis showed gSOS as a novel genetic predictor for fracture risk independent of BMD among breast cancer patients treated with AIs. Future studies are warranted to evaluate the performance of incorporating gSOS in prediction models for the risk of AI-related fracture in breast cancer patients.

## Introduction

Aromatase inhibitors (AIs) are the current endocrine therapy of choice for postmenopausal women diagnosed with hormone receptor (HR)-positive breast cancer, given their superior efficacy over tamoxifen in lowering cancer recurrence. The American Society of Clinical Oncology (ASCO) recommends HR-positive breast cancer patients be offered extended AI therapy beyond the standard five-year course, up to 10 years, based on individualized recurrence risk and treatment tolerability^1^. Because of markedly suppressed aromatase activity and reduced circulating estrogen levels with AI therapy, a major treatment-related effect is bone loss and osteoporotic fracture^2–4^.

Due to their elevated risk of fractures, several medical societies recommend breast cancer patients treated with AIs be assessed for fracture risk using bone mineral density (BMD) testing, mostly dual-energy X-ray absorptiometry (DXA), and clinical risk factors for osteoporosis^5–8^. The Fracture Risk Assessment Tool (FRAX) that combines clinical risk factors and BMD testing can be used to select candidates for osteoporosis treatment^9^, but FRAX was not designed to assess fracture risk in breast cancer patients on AIs and may inaccurately predict estimate risk. Furthermore, long-term monitoring of BMD in breast cancer patients on AIs has not been widely implemented in clinical practice, due to various logistic challenges^10^. Newer fracture risk assessment tools specifically for breast cancer patients who undergo AI treatment are warranted.

In recent years, interest in incorporating genetic predictors for clinical risk assessment has been growing^11^. The maturation of the genome-wide association studies (GWAS) literature has made it possible to compute polygenic scores (PGS) to better capture multi-variant genetic predisposition, compared with a single genetic variant^12–14^. A recent GWAS of heel quantitative ultrasound speed of sound (SOS), which is moderately correlated with BMD, developed a new PGS for SOS (gSOS) based on 21,717 genetic variants that explains 23.2% of the variations of SOS^15^. In a later study, a higher gSOS was linked with lower fracture risk, an association stronger than several clinical factors used by FRAX^16^. It should be noted however that gSOS was developed and tested in populations of European ancestry. Its performance in populations of non-European descent may be inferior due to distinct genetic structure across ethnic groups^17^.

To our knowledge, no studies to date have evaluated gSOS in breast cancer patients treated with AIs, a population at elevated risk of osteoporotic fracture. In a large prospective study of bone health in breast cancer patients on AIs, we analyzed gSOS with risk of major osteoporotic fractures and its dependence on clinical risk factors and BMD measured at the time of cancer diagnosis.

## Results

### Patient descriptive characteristics

Descriptive characteristics of the study population are summarized in Table 1. All women included in these analyses received AI as their initial endocrine therapy for HR-positive breast cancer and had gSOS calculated from genotype data. The median age at diagnosis was 63 (range 28-94) years, with 94% diagnosed after menopause. Self-reported race for most of the study population was White (78%), with about 9% Asian, 6% Black, and 7% Hispanic. The median body mass index (BMI) was 27.9 (range: 15.6-62.0) kg/m^2^; approximately 32% of the women were classified as having an overweight BMI (30 > BMI ≥ 25), with another 40% as having an obese BMI (BMI ≥ 30). Most women were diagnosed with stage I (55%) or II (34%) breast cancer, with 10% stage III, and less than 2% stage IV. In addition to AI therapy, most women also received adjuvant chemotherapy and/or radiation therapy, with 21% receiving endocrine therapy only. The total length of AI treatment varied, with 29% for two years or less, 17% between two and four years, 34% between four and five years, and 21% longer than five years. Only 8% of the study population had a history of osteoporosis, and less than 5% had a history of osteoporotic fracture before breast cancer.

**Table 1:** Characteristics of women with hormone-receptor positive breast cancer who received aromatase inhibitors (AI) and genotype data in the Pathways Study.

Variable	*n* (%)
Age at breast cancer diagnosis, median (range), years	63 (28–94)
Age at breast cancer diagnosis, years, *n* (%)	
<50	69 (3.6)
50–59	551 (28.6)
60–69	815 (42.3)
≥70	492 (25.5)
Menopausal status at breast cancer diagnosis	
Premenopausal	123 (6.4)
Postmenopausal	1804 (93.6)
Race/ethnicity	
White	1499 (77.8)
Black	115 (6.0)
Asian	170 (8.8)
Hispanic	143 (7.4)
Body mass index at breast cancer diagnosis, median (range), kg/m^2^	27.9 (15.6–62.0)
Body mass index at breast cancer diagnosis, kg/m^2^	
<25 kg/m^2^	532 (27.6)
25–29.9 kg/m^2^	618 (32.1)
≥30 kg/m^2^	777 (40.3)
AJCC stage	
I	1058 (54.9)
II	656 (34.0)
III	184 (9.6)
IV	29 (1.5)
Breast cancer treatment	
Chemotherapy	217 (11.3)
Radiation therapy	798 (41.4)
Both	508 (26.4)
None	404 (21.0)
AI treatment length	
≤2 years	558 (29.0)
2.1–4 years	322 (16.7)
4.1–5 years	644 (33.5)
>5 years	401 (20.8)
Bisphosphonate use after AI initiation	
No	1357 (70.4)
Yes	570 (29.6)
Osteoporosis prior to breast cancer diagnosis	
No	1779 (92.3)
<5 years	104 (5.4)
≥5 years	44 (2.3)
Major osteoporotic fracture prior to breast cancer diagnosis	
No	1836 (95.3)
<5 years	57 (3.0)
≥5 years	34 (1.8)
Baseline BMD at the spine, median (range)	0.99 (0.55, 1.68)
Baseline BMD at the hip, median (range)	0.89 (0.45, 1.44)
Baseline BMD at the femoral neck, median (range)	0.73 (0.38, 1.15)

### Correlation between gSOS and BMD

At baseline, 1336 women had BMD measured at the spine, the hip and the femoral neck by DXA scan. Among White women, a weak correlation between gSOS and BMD was apparent at each of the three anatomical sites (correlation coefficients ranged from 0.21-0.23, *p* < 0.0001) (Supplementary Fig. 1).

### Univariate analysis of factors associated with fracture risk

In univariate analyses as summarized in Table 2, older age at diagnosis was significantly associated with elevated risk of fracture. In comparison to White women, Black and Asian women had lower risk of fracture. As expected, women who had a history of osteoporosis and prior fracture were at significantly higher risk of fracture after breast cancer diagnosis, while higher BMD at baseline was associated with lower risk of fracture. When adjuvant therapy modality was considered, women who received AI endocrine therapy only had significantly higher risk of fracture than women who were also treated with chemotherapy and/or radiation therapy. When analyzed as a time-dependent variable, longer than five years of AI treatment was associated with a moderate, non-significant increased risk of fracture compared to those with 2 years or less of AI treatment.

**Table 2:** Univariate associations of demographic and clinical factors with fracture risk in breast cancer patients on aromatase inhibitors (AI).

Variable	# Fracture/total	HR (95% CI)	*P*-value
Age at breast cancer diagnosis, years			<0.001
<50	6/79	1.00	–
50–59	58/639	1.28 (0.55–2.98)	–
60–69	107/890	1.78 (0.78–4.06)	–
≥70	141/544	4.81 (2.12–10.89)	–
Age at breast cancer diagnosis, per year	NA	1.07 (1.06, 1.08)	<0.001
Menopausal status at breast cancer diagnosis			0.01
Premenopausal	11/142	1.00	–
Postmenopausal	301/2010	2.17 (1.19–3.97)	–
Race/ethnicity			0.007
White	248/1565	1.00	–
Black	10/122	0.53 (0.28–1.00)	–
Asian	20/224	0.52 (0.33–0.83)	–
Hispanic	30/199	0.95 (0.65–1.39)	–
Other	4/42	0.58 (0.22–1.56)	–
Body mass index at breast cancer diagnosis, kg/m^2^			0.15
<25.0	94/580	1.00	–
25.0–29.9	108/699	0.94 (0.71–1.24)	–
≥30.0	110/873	0.77 (0.59–1.02)	–
Body mass index at breast cancer diagnosis, kg/m^2^, per unit increase	NA	0.99 (0.97, 1.01)	0.25
AJCC stage			0.71
I	170/1166	1.00	–
II	110/746	1.09 (0.86–1.39)	–
III	29/205	1.20 (0.81–1.78)	–
IV	3/35	1.45 (0.46–4.56)	–
Breast cancer treatment			0.001
Chemotherapy	29/254	1.00	–
Radiation therapy	119/869	1.11 (0.74–1.66)	–
Both	73/572	1.08 (0.70–1.66)	–
None	91/457	1.80 (1.18–2.73)	–
AI treatment length - time-dependent model			0.62
≤2.0 years	NA	1.00	–
2.1–4.0 years	NA	1.14 (0.78, 1.65)	–
4.1–5.0 years	NA	1.11 (0.77, 1.61)	–
>5.0 years	NA	1.30 (0.88, 1.91)	–
AI treatment length, per year - time-dependent model	NA	1.03 (0.97, 1.11)	0.34
Osteoporosis prior to breast cancer diagnosis			<0.001
No	263/1986	1.00	–
Yes	49/165	2.69 (1.98, 3.65)	–
Bisphosphonate use after AI initiation - time-dependent model			
No	1511	1.00	–
Yes	641	2.36 (1.87, 2.99)	<0.001
Bisphosphonate use after AI initiation, per year - time-dependent model		1.15 (1.07, 1.24)	<0.001
Major osteoporotic fracture prior to breast cancer diagnosis			<0.001
No	284/2052	1.00	–
Yes	28/99	2.63 (1.78, 3.88)	–
BMD at the time close to initiation of AI treatment, 0.1 increment			
Spine	NA	0.88 (0.80–0.96)	0.006
Hip	NA	0.70 (0.62–0.79)	<0.001
Femoral neck	NA	0.74 (0.62–0.89)	<0.001
Polygenic score of gSOS			<0.001
Tertile 1	124/636	1.00	–
Tertile 2	83/635	0.63 (0.48, 0.83)	–
Tertile 3	74/656	0.54 (0.41, 0.73)	–
Polygenic score of gSOS, per unit increase	NA	0.52 (0.40, 0.67)	<0.001

To further explore the impact of duration of AI treatment on the association of gSOS with fracture, we compared the PGS, as well as baseline femoral BMD, between those with and without fracture, stratified by duration of AI treatment. Although not statistically significant in all the strata, women with fractures consistently had lower gSOS PGS than those without fracture (Supplementary Table 1). The trend was less consistent for baseline femoral BMD.

### Association of gSOS with fracture risk

**Fig. 1:**
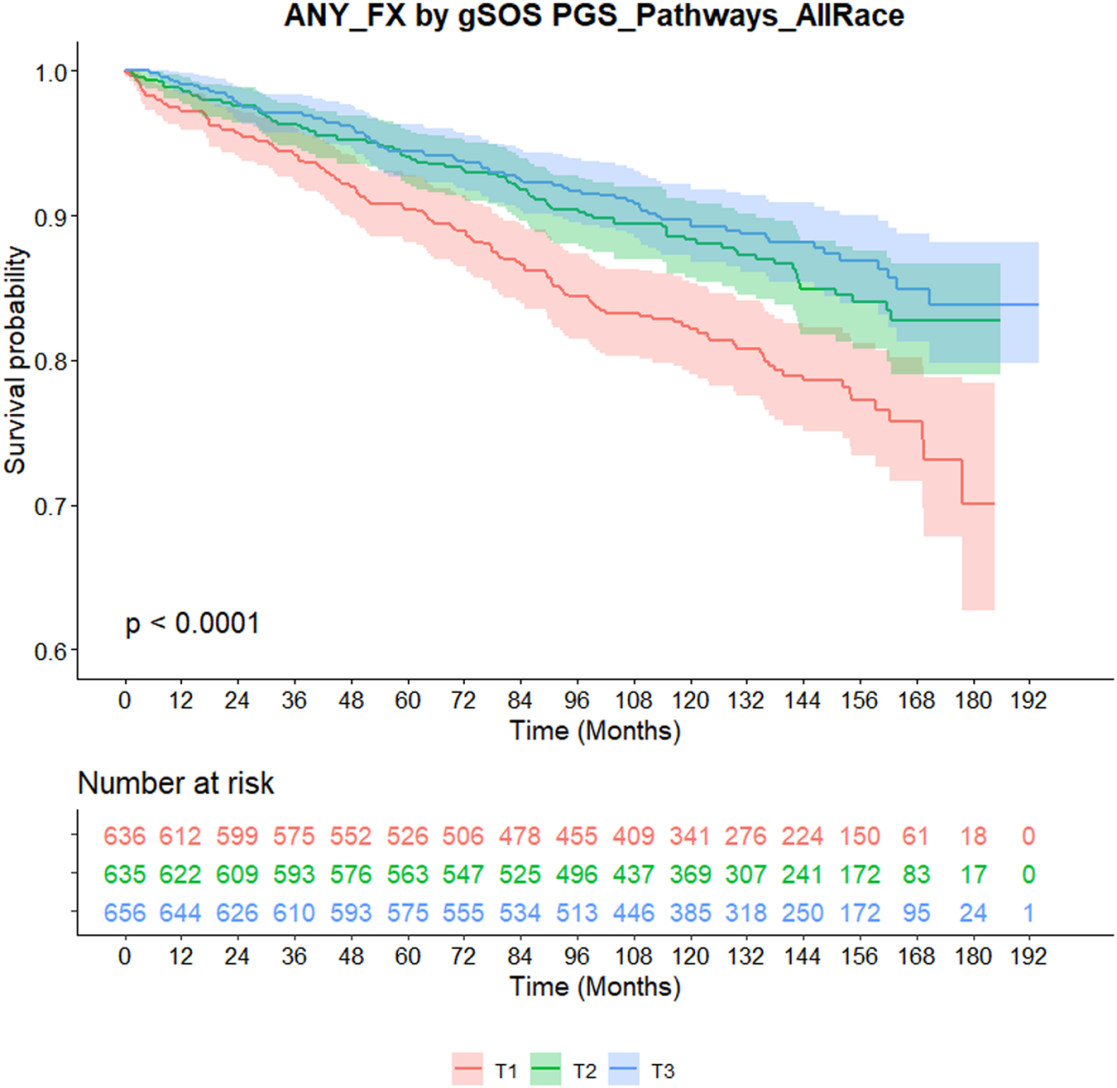
Kaplan-Meier curves of fracture by the tertile of gSOS in breast cancer patients treated with aromatase inhibitors. The probability of staying fracture-free from the beginning of follow up is plotted by the tertiles of gSOS, with corresponding 95% confidence interval shown as shades (T1: red; T2: green; T3: blue). The number of patients at risk at set timepoints are shown in the table below the curves. P-value is derived from log-rank test.

As shown in Kaplan-Meier survival curves in Fig. 1, a clear separation of fracture risk was apparent by the race- or ethnicity-specific tertiles of gSOS in all patients combined (*p* < 0.0001). A higher gSOS was associated with lower risk of fracture, with a significant linear trend (per unit increase, HR = 0.52, 95% CI: 0.40–0.67, *p* = 2.9e-7). When examined within each ethnic group, the differences in fracture risk across gSOS tertiles were statistically significant in Whites, with a similar trend observed in Asians, but not in Blacks or Hispanics (Supplementary Fig. 2).

In multivariable Cox proportional hazards models adjusted for age at diagnosis, patients with the second and third highest tertiles versus the lowest tertile of gSOS had significantly lower risk of fracture (T2: HR = 0.61, 95% CI: 0.47-0.81; T3: HR = 0.53, 95% CI: 0.40–0.71; Table 3). Additional adjustment for ethnicity, cancer treatment, both AI treatment duration and bisphosphonate treatment duration as time-varying variables, and prior history of osteoporosis and fracture did not diminish the associations (T2: HR = 0.61, 95% CI: 0.46-0.80; T3: HR = 0.53, 95% CI: 0.40–0.70). The lower risk of fracture in patients with the highest tertile of PGS remained significant after further adjustment for BMD at the hip in the model (T3: HR = 0.62, 95% CI: 0.42–0.91). Because only approximately 60% of the patients had BMD data at baseline, we restricted the analysis in models 1 and 2 to only patients with hip BMD data. The results are very similar to those without such restriction (Supplementary Table 2). When stratified by ethnicity, the association was significant only among non-Hispanic White patients (Supplementary Table 3). The lack of significance might be attributable to the lower sample size of the minoritized ethnic groups and the fact that the gSOS was initially developed based on population of European descent.

**Table 3:** Multivariable associations of gSOS tertiles with fracture risk in breast cancer patients on aromatase inhibitors (AI).

gSOS PGS	Model 1^a^		Model 2^b^		Model 3^c^	
	HR (95% CI)	*P* trend	HR (95% CI)	*P* trend	HR (95% CI)	*P* trend
gSOS continuous	0.54 (0.42–0.69)	1.76E-06	0.57 (0.44-0.75)	3.35E-05	0.74 (0.53, 1.05)	0.09
Tertile 1	1.00	1.78E-05	1.00	1.64E-05	1.00	3.33E-02
Tertile 2	0.61 (0.47–0.81)	–	0.61 (0.46–0.80)	–	0.71 (0.49–1.02)	–
Tertile 3	0.53 (0.40–0.71)	–	0.53 (0.40–0.70)	–	0.62 (0.42–0.91)	–

^a^Model 1: adjusted for age at diagnosis

^b^Model 2: adjusted for age at diagnosis, prior history of osteoporosis, prior history of major fracture, race and ethnicity, cancer treatment, and AI treatment as a time-varying variable

^c^Model 3: adjusted for the same covariates as in Model 2 plus BMD at the hip.

## Discussion

In a large prospective cohort study of women with breast cancer, we found significant associations of higher gSOS, a polygenic score of heel quantitative ultrasound speed of sound, with lower risk of AI treatment-related fracture that was independent of known clinical predictors of fracture, including BMD at baseline. As gSOS was developed in a general non-cancer population, our study has now demonstrated the predictive value of gSOS for fracture risk in women treated with AIs for breast cancer.

Previous studies have established accelerated loss of BMD and increased risk of fracture in breast cancer patients who receive AI treatment versus those who receive tamoxifen^2–4,18^. However, current risk stratification and clinical management of the population of at-risk breast cancer patients are still extrapolated from postmenopausal women without breast cancer. Considering AI is a potent anti-estrogen agent that is typically prescribed for 5 years and up to 10 years in breast cancer patients, it is of clinical relevance to evaluate whether AI exposure alters the risk factor profile for fracture. The present analysis confirms that the polygenic score developed in the general population is also a strong predictor for risk of fracture in breast cancer patients treated with AIs. This finding, together with our earlier work on demographic, lifestyle, and molecular factors^19–21^, suggests that breast cancer patients treated with AIs and women without breast cancer share similar risk factors for fracture.

Interestingly, we found that the duration of AI treatment for longer than the standard 5-year course was associated with a moderate yet non-significant 30% higher risk of fracture compared to those treated for 2 years or less. The lack of significance could be attributed to suboptimal statistical power. However, among those on AIs for <5 years, little difference in the risk of fracture was observed. Although longer duration of AI treatment has been demonstrated to increase risk of fracture, the data almost always come from comparisons with patients treated with tamoxifen^4^. We are unaware of other studies comparing fracture risk among those who completed the recommended minimum 5 years of AI treatment versus those who discontinued earlier. Patients in our study were outside the clinical trial setting and had a wide range of AI treatment duration. Our data suggest that a full 5-years of AI treatment might not necessarily put patients at higher risk of fracture compared to those with shorter AI courses. These findings warrant validation in future studies.

The lack of difference in fracture risk among those with AI treatment for shorter than 5 years might be due to reverse causality. Those who discontinued AI within 2 years might stop the treatment prematurely due to fracture or rapidly declining BMD, thus they might not be the most appropriate comparison group for those who tolerated AI treatment for a longer time. Similar concerns of reverse causality might also hold true for the unexpected increased risk of fracture among those treated with bisphosphonates. Controlling for the length of AI therapy and bisphosphonate therapy as time-varying variables had essentially no impact on the associations of gSOS with fracture, again suggesting no substantial influence of AI treatment on the associations of known risk factors with fracture in breast cancer patients.

Polygenic risk scores hold promise as a novel avenue to identify and screen at-risk individuals to tailor prophylactic measures to lower morbidity and mortality^11^. In our analyses, although SOS is a bone density measure that is correlated with BMD, gSOS showed independent effects from BMD in association with fracture risk in breast cancer patients treated with AIs. Further, within each AI treatment duration category, gSOS is almost always lower in patients who developed fracture than those who did not, while the differences were less consistent for BMD. Because gSOS can be derived from germline genotype data obtained at any time in a person’s life, which also remains unchanged, gSOS might be more practical than BMD as a predictive tool for fracture risk, as the latter constantly changes and is measured by sophisticated instrumentation such as a DXA scan. The previous study also demonstrated that gSOS can be used as a pre-screening tool to reduce the proportion of patients in need for the more involved clinical risk assessment or BMD measurement^15^.

However, gSOS also comes with some inherent bias due to the original data utilized in the creation of this scoring system. Since most published GWAS to date come from populations of European ancestry, the scores can lead to inaccurate estimation of risk when applied to populations of non-European ancestry^17^. Therefore, we applied the gSOS algorithm and categorized the scores into tertiles separately for each racial and ethnic population. As expected, gSOS was highly associated with fracture risk in White patients, less significant but still in the same direction in Asian patients, and not associated in Black or Hispanic patients, although the sample size was limited in those groups. These race- and ethnicity-specific findings for gSOS were consistent with another study in the general population without cancer^16^. Because much of our patient population were White, the results from analyses of White-only and all groups combined were similar. In future work that may incorporate gSOS or similar PGS into clinical fracture risk prediction models such as FRAX, it would warrant the development of pan-ancestry gSOS based on patient populations of diverse ancestral background, thus the genetic score can be applied regardless of self-reported ethnicity, which is a social construct, inaccurate, and associated with negative connotations.

In conclusion, in this large prospective cohort study of women treated with AIs for breast cancer, the polygenic score, gSOS, was significantly and inversely associated with fracture risk independent of BMD and other fracture risk factors. Development of ancestry-specific gSOS polygenic risk scores is needed to confirm the utility of a gSOS in non-European ancestry populations. Future studies are also recommended to evaluate the performance of incorporating gSOS in prediction models for the risk of AI-related fracture in breast cancer patients.

## Methods

### Study population

The Pathways Study is a prospective cohort of 4,505 women newly diagnosed with invasive breast cancer enrolled between 2006 and 2013 in Kaiser Permanente Northern California (KPNC) after providing written informed consent^19–23^. Non-fasting blood and/or saliva samples were collected as sources of genomic DNA from the patients around the time of the baseline interview. Specimens were shipped to the Data Bank and BioRepository (DBBR) laboratories at Roswell Park Comprehensive Cancer Center for processing and long-term storage. For this study of bone health in breast cancer patients, 1927 patients in the Pathways Study who received both AIs as the initial endocrine therapy, who had genotype data, and who self-identified as non-Hispanic White, non-Hispanic Black, Asian, and Hispanic/Latino, were included. This study was approved by the Institutional Review Boards of Roswell Park Comprehensive Cancer Center and KPNC for compliance with all relevant ethical regulations including the Declaration of Helsinki, and all patients provided their informed consent to participate.

### Clinical and pharmacy data

Tumor characteristics and other data were extracted from the KPNC Virtual Data Warehouse (VDW), which includes data from the KPNC Cancer Registry, pharmacy, encounters, KP electronic health records, and other clinical and administrative data relevant to these analyses. Pharmacy data included outpatient prescriptions for AIs (steroidal exemestane was rarely used in this patient population and most patients received non-steroidal anastrozole, letrozole) and bisphosphonates after breast cancer diagnosis and dates of prescription fills.

### BMD and fracture data

BMD data for the femoral neck, total hip, and lumbar spine were extracted from radiology reports from DXA scans in the KPNC electronic health record (EHR). Algorithms were developed for this purpose, with the performance validated by comparison to manual review as previously reported^24^. Because DXA scans were performed at the discretion of physicians, the timing of the scans relative to cancer diagnosis varied across patients. We defined baseline BMD as those obtained from DXA scans performed within 3 years before or 3 months after breast cancer diagnosis. As a result, a total of 1,336 (62.1%) patients had a baseline BMD. Fractures occurring after the initiation of endocrine therapy up to May 2021 were obtained from the VDW using ICD-9 and ICD-10 diagnosis codes. Fractures that occurred before December 2015 were manually reviewed by a medical record abstractor and subsequently validated by the study endocrinologist (J. Lo)^25^. Non-fragility fractures, including those due to trauma and bone metastases, were excluded from analysis. History of fracture before cancer diagnosis were controlled for in multivariable analysis. Major fragility fractures were defined as those at the humerus, wrist, hip, or spine. Previous history of osteoporosis and major fractures before breast cancer diagnosis were obtained from the VDW as previously described^25^.

### Genotype data and calculation of gSOS

Genomic DNA was extracted from whole blood using commercially available Qiagen QIAamp DNA kits and from saliva samples using Oragene DNA kits following manufacturer’s protocols, which was used for subsequent genotyping work^26^. In brief, the Illumina Multi-Ethnic Global Array (MEGA) with inclusion of custom content. Following sample- and marker-level QC, imputation was conducted using the University of Michigan Imputation Server and the Haplotype Reference Consortium (HRC) reference panel. Poorly imputed variants with imputation quality R-square <0.3 were excluded. For computation of gSOS, the list of 21,716 genetic variants and associated weights were downloaded from the Polygenic Score (PGS) Catalog database (PGS000657)^27^. gSOS used LASSO models to predict SOS using only SNPs with p-values smaller than a chosen set of thresholds that resulted in the lowest root mean square error for the prediction of SOS and thus not all variants chosen reached genome-wide significance. Also, the weights for the variants were not race/ethnicity specific, because the original study was performed only in White British individuals but not in a multi-ethnic group. A total of 14,491 variants were matched with the imputed data from Pathways and used to calculate PGS separately for each of four main self-reported racial and ethnic groups, including Asian, Hispanic, non-Hispanic Black (NHB), and non-Hispanic White (NHW). The raw PGS was analyzed either as numeric values or categorized into equal tertiles (T) based on the distribution within each racial and ethnic group. The fewer variants included in the calculation of gSOS might have led to less accurate prediction of SOS or subsequently risk of fracture. However, we were unable to evaluate these scenarios due to lack of SOS phenotype data from our study. We anticipated the impact to be relatively minor.

### Statistical analysis

For descriptive analyses, count (percent) and median (range) were summarized. Kaplan-Meier curves by the tertiles of gSOS were generated for each racial and ethnic group separately, as well as with all groups combined, with p-values derived from log-rank tests. Correlations of gSOS with baseline BMD at the hip, the spine and the femoral head were plotted and examined using the Pearson correlation test. Univariate analyses of demographic and clinical factors with fracture risk were conducted using Cox proportional hazards models. AI duration was examined as a time-varying variable in Cox models for 2.1 to 4 years, 4.1–5 years, and longer than 5 years, in comparison to 2 years or less. To further explore the impact of AI duration on fracture risk, the length of treatment was grouped at a yearly increase and the associations of gSOS and baseline BMD with fracture were examined within each group of AI duration using univariate Cox regression. Three multivariable Cox regression models of fracture risk with tertiles of gSOS were conducted, with adjustment for age at diagnosis only, all covariates significant in univariate analysis plus AI duration as a time-varying covariate, and the former plus baseline BMD at the hip. No violation of proportional hazards assumptions were found by examining plots of the scaled Schoenfeld residuals. All analyses were conducted using R 4.2.0.

### Reporting summary

Further information on research design is available in the Nature Research Reporting Summary linked to this article.

### Supplementary information

Supplemental Materials

Reporting Summary

## Data Availability

The genotype-wide genotype data of Pathways participants have already been deposited in dbGaP (access number: phs001534.v1.p1). The fracture data used in the current study are available from the corresponding author on reasonable request.

## Code availability

All analyses were performed in R programing using base R and survival packages. The analytical codes are available from the corresponding author upon reasonable request.
