# Supplementary material for: A polygenic score associated with fracture risk in breast cancer patients treated with aromatase inhibitors
Source: NPJ Breast Cancer. 2024 Jan 20;10:9. doi: 10.1038/s41523-024-00615-9 (PMC10799916; doi:10.1038/s41523-024-00615-9)

Supplementary Table 1. gSOS and femoral bone mineral density by duration of aromatase inhibitor (AI) treatment

AI Duration	# Fracture/non-fracture	PGS, mean (std)			Femoral BMD, mean (std)		
		Fracture	Non-fracture	P-value*	Fracture	Non-fracture	P-value*
≤1.0 year	73/319	-0.59 (0.5)	-0.47 (0.45)	0.07	0.69 (0.12)	0.76 (0.12)	0.008
1.1-2.0 years	47/191	-0.57 (0.47)	-0.43 (0.45)	0.08	0.71 (0.11)	0.74 (0.13)	0.42
2.1-3.0 years	34/128	-0.56 (0.4)	-0.53 (0.51)	0.73	0.76 (0.14)	0.72 (0.12)	0.46
3.1-4.0 years	35/166	-0.69 (0.32)	-0.41 (0.44)	0.0002	0.65 (0.11)	0.78 (0.15)	0.01
4.1-5.0 years	73/638	-0.56 (0.47)	-0.43 (0.47)	0.04	0.69 (0.09)	0.75 (0.12)	0.008
5.1-6.0 years	30/235	-0.56 (0.43)	-0.43 (0.46)	0.13	0.77 (0.1)	0.76 (0.13)	0.74
>6.0 years	18/163	-0.73 (0.35)	-0.40 (0.42)	0.005	0.84 (0.09)	0.74 (0.11)	0.36

Footnote: * P-values derived from log-rank test of binary PGS (high vs. low by median) with fracture.

Supplementary Table 2. Multivariable associations of gSOS tertiles with fracture risk in breast cancer patients on aromatase inhibitors (AI) among patients with baseline bone mineral density data

gSOS PGS	Model 1*		Model 2†		Model 3‡	
	HR (95% CI)	P trend	HR (95% CI)	P trend	HR (95% CI)	P trend
Tertile 1	1.00	0.004	1.00	0.004	1.00	0.03
Tertile 2	0.67 (0.46-0.96)		0.66 (0.46-0.95)		0.71 (0.49-1.02)	
Tertile 3	0.54 (0.37-0.79)		0.54 (0.37-0.79)		0.62 (0.42-0.91)	

* Model 1: Adjusted for age at diagnosis
† Model 2: Adjusted for age at diagnosis, prior history of osteoporosis, prior history of major fracture, race and ethnicity, cancer treatment, and AI treatment as a time-varying variable
‡ Model 3: Adjusted for the same covariates as in Model 2 plus BMD at the hip.

Supplementary Table 3. Multivariable associations of gSOS tertiles with fracture risk in breast cancer patients on aromatase inhibitors (AI) stratified by self-reported ethnicity

gSOS PGS	Model 1* HR (95% CI)	P-value	Model 2† HR (95% CI)	P-value	Model 3‡ HR (95% CI)	P-value
			Non-Hispanic White			
T1	1.00	9.48E-06	1.00	1.23E-05	1.00	0.01
T2	0.59 (0.44, 0.79)		0.58 (0.43, 0.79)		0.68 (0.46, 0.99)	
T3	0.49 (0.36, 0.67)		0.49 (0.36, 0.68)		0.54 (0.36, 0.83)	
			Non-Hispanic Black			
T1	1.00	0.48	1.00	0.51	1.00	0.85
T2	1.18 (0.16, 8.47)		1.14 (0.13, 10.3)		1.71 (0.07, 39.39)	
T3	2.42 (0.47, 12.49)		2.37 (0.43, 12.91)		1.9 (0.2, 18.36)	
			Asian			
T1	1.00	0.13	1.00	0.20	1.00	na
T2	0.62 (0.17, 2.23)		0.48 (0.11, 2.12)		na	
T3	0.16 (0.02, 1.36)		0.17 (0.02, 1.59)		na	
			Hispanic			
T1	1.00	0.81	1.00	0.75	1.00	0.64
T2	0.71 (0.24, 2.13)		0.72 (0.23, 2.24)		0.49 (0.04, 5.62)	
T3	0.92 (0.33, 2.56)		0.63 (0.18, 2.19)		1.39 (0.26, 7.51)	

* Model 1: Adjusted for age at diagnosis
† Model 2: Adjusted for age at diagnosis, prior history of osteoporosis, prior history of major fracture, race and ethnicity, cancer treatment, and AI treatment as a time-varying variable

‡ Model 3: Adjusted for the same covariates as in Model 2 plus BMD at the hip.

Supplementary Figure 1. Correlation plot of gSOS and baseline bone mineral density at the time close to the initiation of aromatase inhibitor (AI) treatment

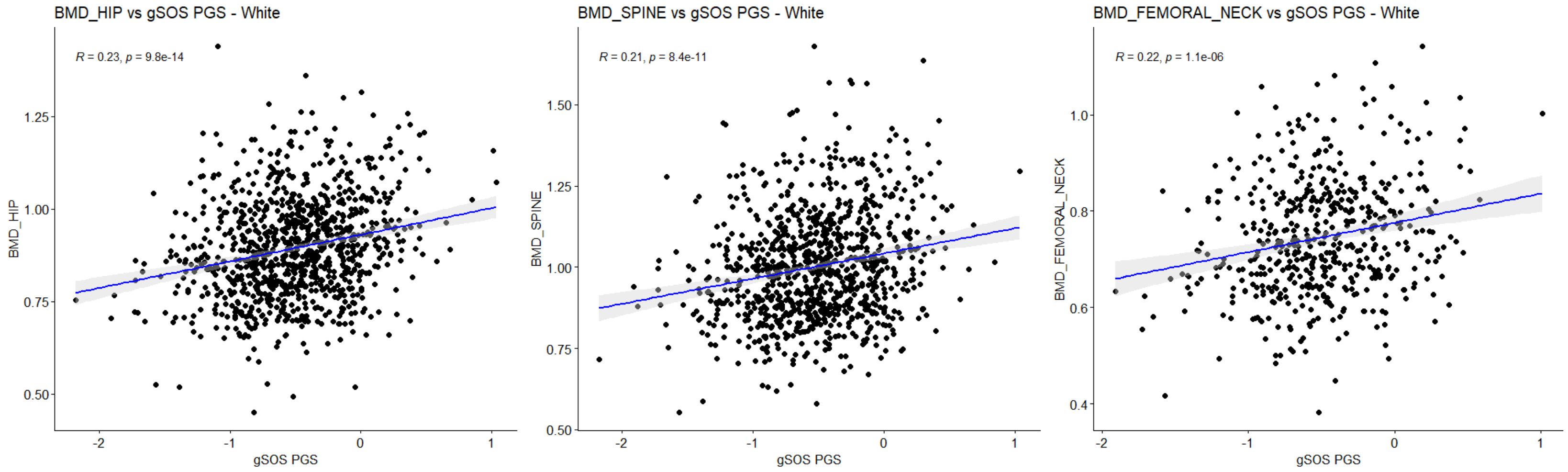

Supplementary Figure 2. Kaplan-Meier curves of fracture by tertile of gSOS in breast cancer patients treated with aromatase inhibitors by race and ethnicity

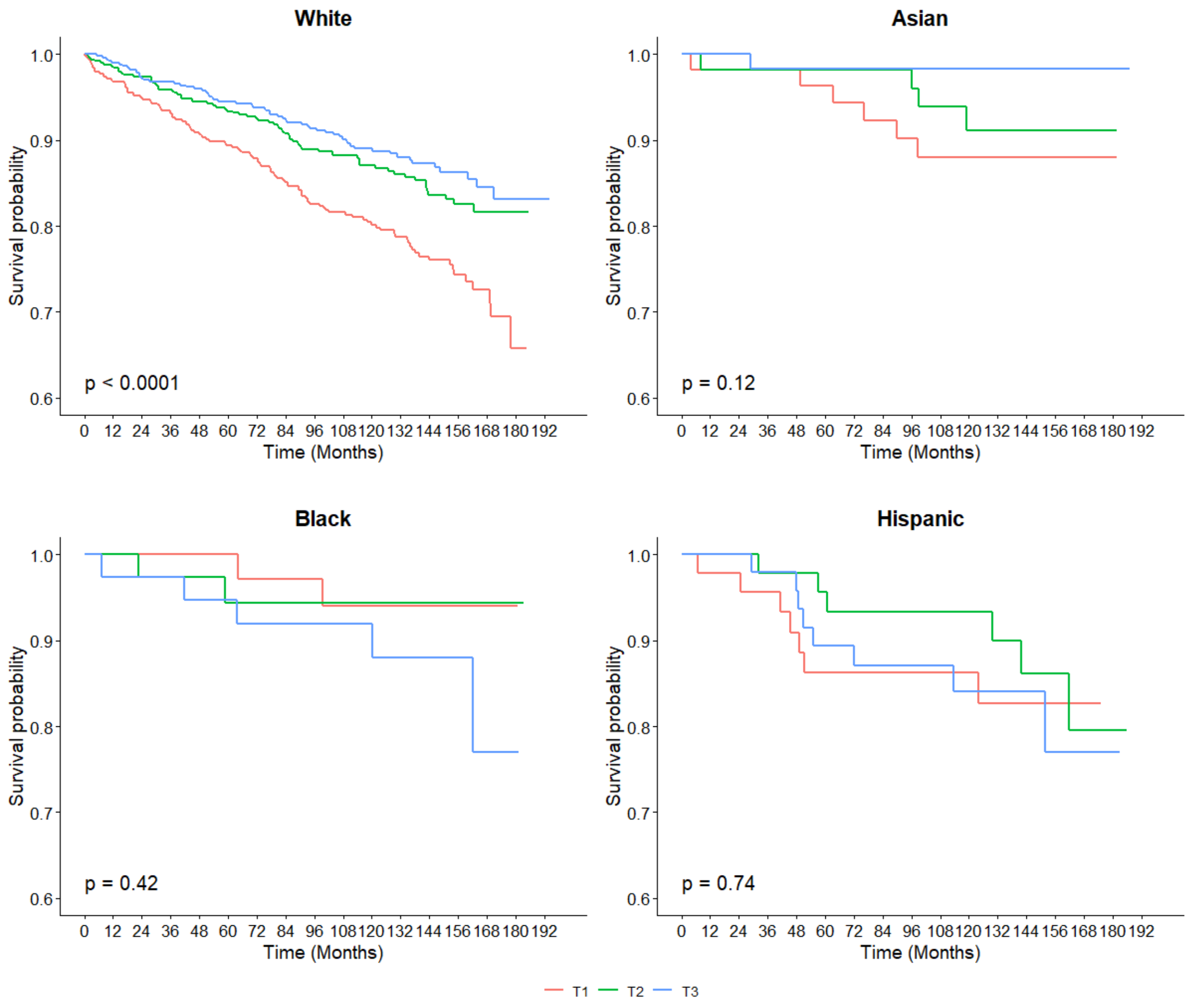

Supplement: Supplementary file 1 — Supplemental Materials [file 41523_2024_615_MOESM1_ESM.pdf]
