# Supplementary material for: A polygenic score associated with fracture risk in breast cancer patients treated with aromatase inhibitors
Source: NPJ Breast Cancer. 2024 Jan 20;10:9. doi: 10.1038/s41523-024-00615-9 (PMC10799916; doi:10.1038/s41523-024-00615-9)
Supplement: Supplementary file 2 — Reporting Summary [file 41523_2024_615_MOESM2_ESM.pdf]

nature portfolio

Corresponding author(s): Song Yao

Last updated by author(s): 01/03/2024

Reporting Summary

Nature Portfolio wishes to improve the reproducibility of the work that we publish. This form provides structure for consistency and transparency in reporting. For further information on Nature Portfolio policies, see our Editorial Policies and the Editorial Policy Checklist.

Statistics

For all statistical analyses, confirm that the following items are present in the figure legend, table legend, main text, or Methods section.

n/a	Confirmed	
☐	☒	The exact sample size (*n*) for each experimental group/condition, given as a discrete number and unit of measurement
☒	☐	A statement on whether measurements were taken from distinct samples or whether the same sample was measured repeatedly
☐	☒	The statistical test(s) used AND whether they are one- or two-sided<br>*Only common tests should be described solely by name; describe more complex techniques in the Methods section.*
☐	☒	A description of all covariates tested
☐	☒	A description of any assumptions or corrections, such as tests of normality and adjustment for multiple comparisons
☐	☒	A full description of the statistical parameters including central tendency (e.g. means) or other basic estimates (e.g. regression coefficient) AND variation (e.g. standard deviation) or associated estimates of uncertainty (e.g. confidence intervals)
☐	☒	For null hypothesis testing, the test statistic (e.g. *F*, *t*, *r*) with confidence intervals, effect sizes, degrees of freedom and *P* value noted<br>*Give P values as exact values whenever suitable.*
☒	☐	For Bayesian analysis, information on the choice of priors and Markov chain Monte Carlo settings
☒	☐	For hierarchical and complex designs, identification of the appropriate level for tests and full reporting of outcomes
☒	☐	Estimates of effect sizes (e.g. Cohen's *d*, Pearson's *r*), indicating how they were calculated

Our web collection on statistics for biologists contains articles on many of the points above.

Software and code

Policy information about availability of computer code

Data collection	*Provide a description of all commercial, open source and custom code used to collect the data in this study, specifying the version used OR state that no software was used.*
Data analysis	R Programming 4.2.0

For manuscripts utilizing custom algorithms or software that are central to the research but not yet described in published literature, software must be made available to editors and reviewers. We strongly encourage code deposition in a community repository (e.g. GitHub). See the Nature Portfolio guidelines for submitting code & software for further information.

Data

Policy information about availability of data

All manuscripts must include a data availability statement. This statement should provide the following information, where applicable:

- Accession codes, unique identifiers, or web links for publicly available datasets
- A description of any restrictions on data availability
- For clinical datasets or third party data, please ensure that the statement adheres to our policy

The genotype-wide genotype data of Pathways participants have already been deposited in dbGaP (access number: phs001534.v1.p1). The fracture data used in the current study are available from the corresponding author on reasonable request.

Research involving human participants, their data, or biological material

Policy information about studies with human participants or human data. See also policy information about sex, gender (identity/presentation), and sexual orientation and race, ethnicity and racism.

Reporting on sex and gender	This is study of breast cancer in women.
Reporting on race, ethnicity, or other socially relevant groupings	Race and ethnicity were self-reported by patients when answering questionnaires.
Population characteristics	Descriptive characteristics of the study population are summarized in Table 1. All women included in these analyses received AI as their initial endocrine therapy for HR-positive breast cancer and had gSOS calculated from genotype data. The median age at diagnosis was 63 (range 28-94) years, with 94% diagnosed after menopause.
Recruitment	Participants for Pathways Study were recruited from women who were newly diagnosed with breast cancer at Kaiser Permanente Northern California by screening electronic medical records and informed consent.
Ethics oversight	Roswell Park Comprehensive Cancer Center; Kaiser Permanente Northern California

Note that full information on the approval of the study protocol must also be provided in the manuscript.

Field-specific reporting

Please select the one below that is the best fit for your research. If you are not sure, read the appropriate sections before making your selection.

☒ Life sciences ☐ Behavioural & social sciences ☐ Ecological, evolutionary & environmental sciences

For a reference copy of the document with all sections, see nature.com/documents/nr-reporting-summary-flat.pdf

Life sciences study design

All studies must disclose on these points even when the disclosure is negative.

Sample size	The Pathways Study is a prospective cohort of 4,505 women newly diagnosed with invasive breast cancer enrolled between 2006 and 2013 in Kaiser Permanente Northern California (KPNC) after providing written informed consent. For this study of bone health in breast cancer patients, 1,927 patients in the Pathways Study who received both AIs as the initial endocrine therapy, who had genotype data, and who self-identified as non-Hispanic White, non-Hispanic Black, Asian, and Hispanic/Latino, were included.
Data exclusions	n/a
Replication	n/a
Randomization	n/a
Blinding	n/a

Reporting for specific materials, systems and methods

We require information from authors about some types of materials, experimental systems and methods used in many studies. Here, indicate whether each material, system or method listed is relevant to your study. If you are not sure if a list item applies to your research, read the appropriate section before selecting a response.

Materials & experimental systems

n/a	Involved in the study	
☒	☐	Antibodies
☒	☐	Eukaryotic cell lines
☒	☐	Palaeontology and archaeology
☒	☐	Animals and other organisms
☒	☐	Clinical data
☒	☐	Dual use research of concern
☒	☐	Plants

Methods

n/a	Involved in the study	
☒	☐	ChIP-seq
☒	☐	Flow cytometry
☒	☐	MRI-based neuroimaging
